# Addition of a short HIV-1 fusion-inhibitory peptide to PRO 140 antibody dramatically increases its antiviral breadth and potency

**DOI:** 10.1128/jvi.02018-24

**Published:** 2025-03-25

**Authors:** Hongxia Yan, Yue Gao, Yuanmei Zhu, Huihui Chong, Yani Gong, Yue Chen, Li Li, Bin Su, Yuxian He

**Affiliations:** 1 NHC Key Laboratory of Systems Biology of Pathogens, National Institute of Pathogen Biology, Chinese Academy of Medical Sciences and Peking Union Medical College 220736 , Beijing, China; 2 Beijing Key Laboratory for HIV/AIDS Research, Clinical and Research Center for Infectious Diseases, Beijing Youan Hospital, Capital Medical University 194045 , Beijing, China; 3 Center for AIDS Research, Chinese Academy of Medical Sciences and Peking Union Medical College 12501 , Beijing, China; University Hospital Tübingen, Tübingen, Germany

**Keywords:** HIV-1, CCR5, PRO 140, fusion inhibitor

## Abstract

PRO 140, a humanized anti-HIV monoclonal antibody targeting the cell coreceptor CCR5, is currently under clinical trials, but it only affects CCR5-tropic viruses. In this study, we have engineered two tandem fusion proteins (2P23-PRO140SC and 2P23-PRO140-Fc) with bifunctional activity by adding short fusion-inhibitory peptide 2P23 to the single-chain fragment variable (scFv) of PRO 140 (PRO140SC) with or without the Fc domain of human IgG4. We first demonstrated that 2P23-PRO140SC and 2P23-PRO140-Fc could efficiently bind to the cell membranes through CCR5 anchoring, which did not affect the expression level of CCR5 on the cell surface. We then verified that the addition of 2P23 peptide to PRO140SC enabled a very potent activity against CXCR4-tropic HIV-1 isolates. As expected, the bispecific fusion proteins exhibited highly potent activities in inhibiting divergent HIV-1 subtypes and viral mutants that were resistant to the fusion inhibitors 2P23 and T20, and they displayed relatively low *in vitro* cytotoxicity. Furthermore, both the fusion proteins had robust *in vivo* anti-HIV activities in rats, with 2P23-PRO140-Fc much better than 2P23-PRO140SC. In conclusion, our studies have provided bispecific HIV-1 inhibitors that overcome the drawbacks of PRO 140 antibody and offered novel tools for studying the mechanisms of HIV-1 infection.

**IMPORTANCE:**

Given that HIV-1 evolves with high variability and drug resistance, the development of novel antivirals is important. CCR5-directed antibody PRO 140 is currently under clinical trials, but it only inhibits CCR5-tropic HIV-1 isolates. The designed fusion proteins by adding a minimum fusion-inhibitory peptide to PRO 140 enable dramatically increased activities in inhibiting both CCR5-tropic and CXCR4-tropic viruses, thus offering novel antiviral agents with a bispecific functionality that can overcome the drawbacks of PRO 140 antibody.

## INTRODUCTION

Combination antiretroviral therapy (cART) with multiple drugs can effectively inhibit HIV-1 replication, but it is unable to eradicate the integrated virus in the host cells (1). Despite the fact that HIV-1 viremia can be durably suppressed, viral load will rapidly rebound if cART is stopped, even in patients harboring very small viral reservoirs and minimal ongoing viral transcription (2). The lifelong cART treatment is often associated with side effects and drug resistance. Therefore, safe and effective approaches are demanded to curtail the HIV-1 epidemic, and novel antiretrovirals are fundamentally important. Given that current antiretroviral classes mainly block one of the viral enzymes, including nucleoside and non-nucleoside reverse transcriptase inhibitors, integrase strand transfer inhibitors, and protease inhibitors, the development of inhibitors that can block HIV-1 entry step, namely HIV-1 entry inhibitors, is one of the top priorities in the research field.

HIV-1 cell entry is mediated by its envelope glycoproteins composed of surface subunit gp120 and transmembrane subunit gp41 (3). While gp120 is responsible for binding with the cell receptor CD4 and coreceptor CCR5 or CXCR4, gp41 mediates the fusion of the viral and cell membranes, and both subunits undergo a cascade of conformational changes during the actions (4). According to the coreceptor usage during infection, HIV-1 strains are classified into CCR5-tropic (R5), CXCR4-tropic (R4), and dual-tropic (R5X4) viruses. Given the fact that the R5 virus plays pivotal roles in the early stages of infection and during sexual transmission, CCR5 has been considered an ideal drug target since its discovery as the primary coreceptor for HIV-1 entry in 1996 (5, 6). Maraviroc (MVC) is a small-molecule CCR5 antagonist that acts by binding to the hydrophobic pocket formed by seven transmembrane helices of CCR5; thus, it inhibits HIV entry via an allosteric mechanism (7, 8). MVC was clinically approved in 2007, but it remains the only available anti-HIV drug targeting CCR5, despite tremendous efforts in the past decades. There are many works devoted to the development of anti-CCR5 monoclonal antibodies (mAbs), as exemplified by PRO 140 (Leronlimab), HGS004, and RoAb13 (9, 10). Different from the mechanism of action by MVC, PRO 140 is a humanized IgG4 anti-CCR5 mAb through competitive binding to the N-terminal and the extracellular loop 2 domains of CCR5, which does not influence the chemokine receptor activity of CCR5 (11–13). It was also observed that PRO 140 and MVC had a potent synergy in inhibiting HIV-1 infection (14, 15). Since PRO 140 was granted the “Fast-Track” designation by the U.S. Food and Drug Administration (FDA) in 2006 as a combination therapy for HIV patients, its potent and prolonged antiretroviral activity as a monotherapy has been evaluated by multiple clinical studies (16–19). However, like MVC and other CCR5 antagonists, PRO 140 is only effective against the R5 virus but not the R4 and R5X4 viruses, and a tropism assay is a pre-requisite prior to the treatment. Therefore, it is desirable to optimize PRO 140 to overcome the limitations.

Because HIV evolves with high variability and drug resistance, bispecific or multi-specific antiretrovirals targeting different steps of HIV-1 entry have been extensively exploited (1, 3). In this track, our studies are committed to developing HIV-1 fusion and multifunctional inhibitors with potent, broad-spectrum activity (20–25). Among them, a helical short-peptide (23-mer) fusion inhibitor 2P23 was rationally designed by introducing the M-T hook structure and salt bridge-forming residues into a mixed peptide sequence of HIV-1, HIV-2, and simian immunodeficiency virus (SIV) (25). 2P23 and its lipid-conjugated derivative LP-19 exhibited very potent, broad-spectrum *in vitro* anti-HIV activities and *in vivo* therapeutic efficacies (25, 26). Very recently, we created a bispecific HIV-1 entry inhibitor termed 2P23-iMab by adding 2P23 peptide to the single-chain fragment variable (scFv) of CD4-targeting antibody ibalizumab (iMab), which had dramatically increased potency and breadth against divergent HIV-1 isolates and those not being susceptible to ibalizumab (27). This promising hit prompted us to develop superior bispecific inhibitors that can simultaneously target the coreceptor CCR5 and viral fusion protein gp41. In this study, we have engineered two tandem fusion proteins (2P23-PRO140SC and 2P23-PRO140-Fc) by adding 2P23 peptide to the scFv of PRO 140 antibody, enabling the dramatically increased antiviral breadth and potency against both CCR5-tropic and CXCR4-tropic HIV-1 isolates. An invention patent for the molecules has been authorized in China (ZL202210438141.6). Herein, we report their design strategies and the results of functional characterizations.

## RESULTS

### Generation of bispecific inhibitors targeting CCR5 and gp41

**Fig 1:**
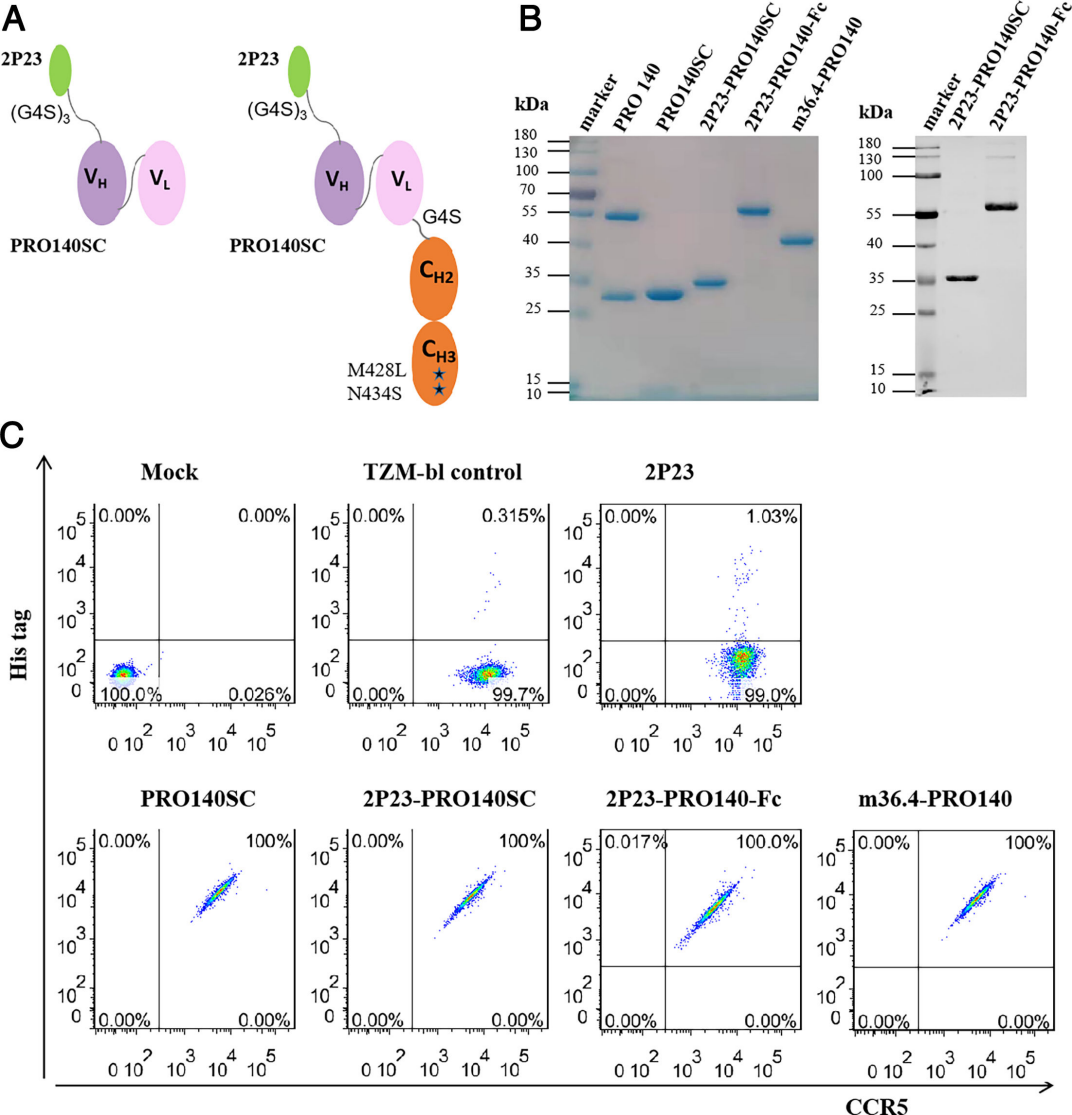
Design and characterization of bispecific HIV-1 inhibitors. (**A**) Schematic diagrams show the structures of 2P23-PRO140SC and 2P23-PRO140-Fc. 2P23 peptide is marked in green. VH represents the heavy chain variable fragment of PRO 140, VL represents the light chain variable fragment of PRO 140, and CH2-CH3 represents the crystallizable fragment (Fc) of human IgG4 with M428L/N434S mutations. (**B**) SDS-PAGE and western blot analysis of PRO 140-based inhibitors. The purity and size of recombinant proteins were analyzed by SDS-PAGE gel (left), and the specificity of 2P23-PRO140SC and 2P23-PRO140-Fc was determined by Western blot analysis with a mouse anti-2P23 peptide antibody 5F7 (right). (**C**) Bindings of PRO 140-based inhibitors and 2P23 peptide with the cell membrane determined by flow cytometry. TZM-bl cells were preincubated with an inhibitor at 4°C for 1 h. After thorough washing, the binding ability of the inhibitor was determined by mouse anti-His tag and anti-CCR5 antibodies. The fluorescence intensities of membrane-bounded inhibitors were quantitated with a FACSCanto II instrument. Mock, TZM-bl cells only; TZM-bl control, the cells were similarly treated but with an inhibitor only. His tag, cells stained with a mouse anti-His tag antibody followed by an Alexa Fluor 488 rabbit anti-mouse IgG antibody. CCR5, cells stained with an APC mouse anti-human CD195 antibody.

Considering that the cell coreceptor CCR5 is an ideal drug target, but its antibody drug PRO 140 only affects R5 HIV-1 strains, we endeavor to develop bispecific HIV entry inhibitors that can simultaneously target CCR5 and viral fusion protein gp41. Herein, two fusion proteins were designed and characterized (Fig. 1A). The first fusion protein 2P23-PRO140SC was created by linking the fusion-inhibitory peptide 2P23 to the scFv of PRO 140 (PRO140SC) in a tandem format. 2P23-PRO140SC was further fused to the N-terminal site of IgG4 Fc domain to generate the second fusion protein 2P23-PRO140-Fc. A full-length PRO 140 antibody prototype, PRO140SC and a previously reported bispecific fusion protein m36.4-PRO140 were also constructed and used as control inhibitors. Recombinant protein inhibitors were expressed in HEK293T cells and purified by affinity chromatography. As analyzed by SDS-PAGE, all the proteins showed high purity and correct size (Fig. 1B, left). Western blot analysis with a mouse anti-2P23 peptide mAb (5F7) verified the specificity of bispecific 2P23-PRO140SC and 2P23-PRO140-Fc (Fig. 1B, right).

The binding abilities of PRO 140-based inhibitors and 2P23 peptide to cells that express CCR5 were characterized by a flow cytometry assay. Each inhibitor was preincubated with TZM-bl cells and washed thoroughly, and the binding of inhibitor was then detected by anti-His tag and anti-human CD195 (CCR5) antibodies. As shown in Fig. 1C, four PRO-140-based inhibitors (PRO140SC, 2P23-PRO140SC, 2P23-PRO140-Fc, and m36.4-PRO140) at a saturated concentration of 100 µg/mL bound to the cell membrane efficiently, with the percentages of anti-His tag and CCR5-double-positive cells being 100%. When tested at different inhibitor concentrations, the bindings of PRO140SC, 2P23-PRO140SC, and 2P23-PRO140-Fc showed dose-dependent effects, which did not affect the expression level of CCR5 on the cell surface (Fig. S1). These results suggest that PRO 140-based inhibitors can efficiently bind to the cell membrane through CCR5 anchoring.

### Addition of 2P23 peptide enables PRO 140 to be highly effective against CXCR4-tropic HIV-1 isolates

**Fig 2:**
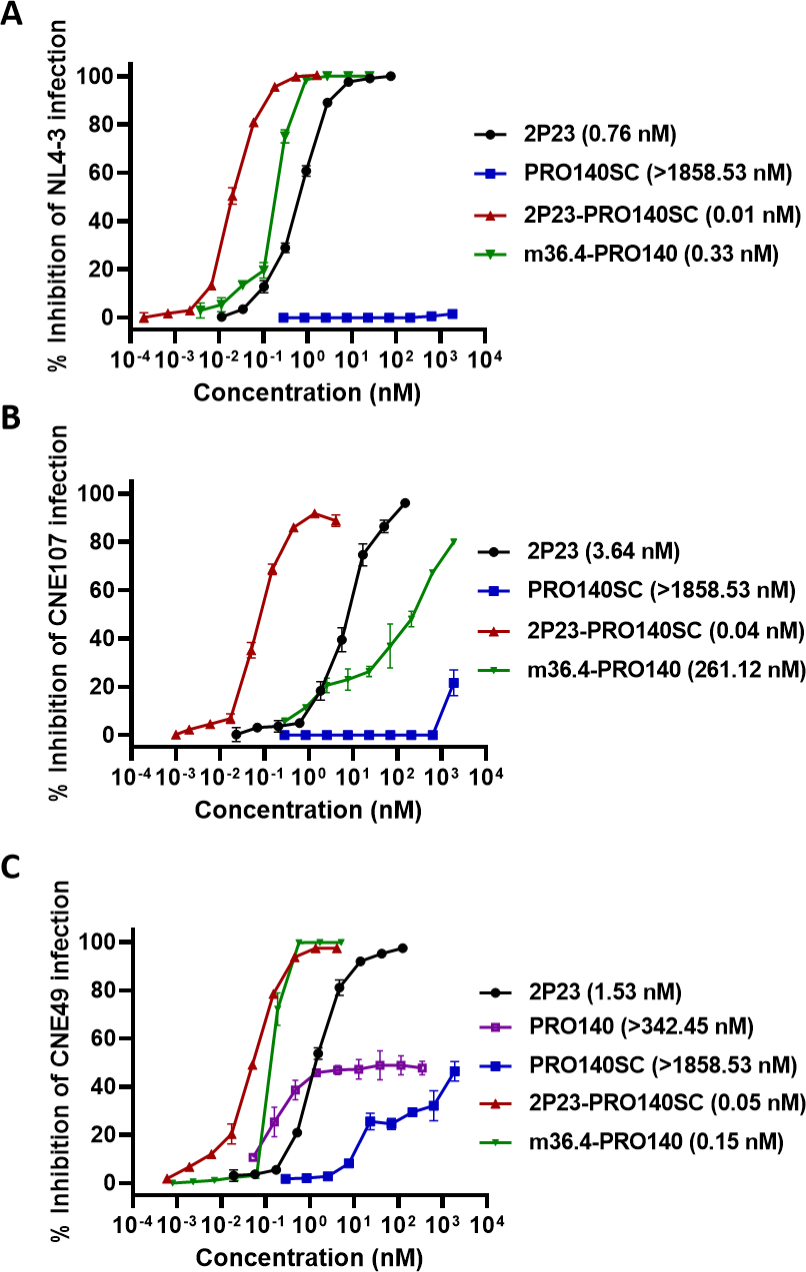
Inhibitory activities of PRO 140-based inhibitors against PRO 140-resistant HIV-1 isolates. The activities of PRO 140-based inhibitors along with 2P23 peptide in inhibiting HIV-1 NL4-3 (**A**), CNE107 (**B**), and CNE49 (**C**) pseudoviruses were respectively determined by a single-cycle infection assay. The assays were performed in triplicate and repeated three times, and data are expressed as mean ± standard deviation (SD).

Given that the PRO 140 antibody targets the cell coreceptor CCR5, thereby inhibiting R5 HIV-1 isolates only, we were eager to know the inhibitory activity of 2P23-PRO140SC against CXCR4-tropic HIV-1 isolates. To this end, two X4 viruses, NL4-3 and CNE107, were used in a single-cycle infection assay. As anticipated in Fig. 2A and B, PRO140SC had no appreciable activity in inhibiting the infections of NL4-3 and CNE107 pseudoviruses at a high concentration (1,858.53 nM), whereas 2P23 peptide inhibited the two viruses with IC_50_ values of 0.76 and 3.64 nM, respectively. In sharp contrast, 2P23-PRO140SC inhibited NL4-3 and CNE107 with dramatically increased potency, as indicated by their IC_50_ values of 0.01 and 0.04 nM, respectively, which were about 76-fold and 91-fold more potent than 2P23 peptide. As a control inhibitor, m36.4-PRO140 inhibited the two viruses with IC_50_ values of 0.33 and 261.12 nM, respectively, which were 33-fold and 6,528-fold less potent than 2P23-PRO140SC. In addition, we identified an R5 virus (CNE49) highly resistant to PRO 140 antibody, but it showed a high susceptibility to the inhibitions of 2P23-PRO140SC and m36.4-PRO140 (Fig. 2C). These results demonstrated that the addition of the 2P23 peptide enabled PRO 140 to be highly effective against CXCR4-tropic HIV-1 isolates and those R5 viruses insensitive to PRO 140 antibody.

Meanwhile, we also determined the synergistic effects between 2P23 and PRO140SC in comparison with the potency of 2P23-PRO140SC. To do so, 2P23 and PRO140SC were mixed at an indicated molar ratio, and their inhibitory activities in combination or themselves alone were determined by the pseudovirus-based infection assay. As shown in Fig. S2, 2P23 and PRO140SC displayed synergistic effects against HIV-1 JRFL and CH70.1 infections with combination index (CI) values of 0.308 and 0.455, respectively. However, the antiviral activities of the two inhibitors in combination were much lower than those of 2P23-PRO140SC.

### Bispecific inhibitors exhibit potent, broad-spectrum antiviral activities against divergent HIV-1 subtypes

We next sought to characterize the antiviral potency and breadth of PRO 140-based inhibitors along with 2P23 peptide. First, the so-called “global panel” HIV-1 Envs, which were derived from divergent HIV-1 subtypes representing the AIDS epidemic worldwide (28), were used to prepare pseudoviruses, and a single-cycle infection assay was conducted. As shown in Table 1, 2P23, PRO 140, and PRO140SC inhibited the global panel viruses with mean IC_50_ values of 3.118, 7.28, and 18.33 nM, respectively; whereas 2P23-PRO140SC and 2P23-PRO140-Fc inhibited these panel viruses with mean IC_50_ values of 0.041 and 0.235 nM, respectively. In comparison, 2P23-PRO140SC was 76-fold more potent than 2P23, 178-fold more potent than PRO 140, and 447-fold more potent than PRO140SC, whereas 2P23-PRO140-Fc exhibited a 5.7-fold decreased activity relative to 2P23-PRO140SC. As a control, m36.4-PRO140 inhibited 12 viruses with a mean IC_50_ at 1.344 nM, indicating a 33-fold decreased potency than 2P23-PRO140SC.

**TABLE 1:** Inhibitory activities of PRO140-based inhibitors and 2P23 peptide against divergent HIV-1 subtypes^*a*^

Pseudovirus	Subtype	Tropism	Mean IC_50_ ± SD (nM)					
			2P23	PRO 140	PRO140SC	2P23-PRO140SC	2P23-PRO140-Fc	m36.4-PRO140
398-F1_F6_20	A	CCR5	2.061 ± 0.418	0.714 ± 0.273	38.209 ± 17.760	0.024 ± 0.002	0.175 ± 0.053	1.803 ± 0.142
TRO.11	B	CCR5	4.351 ± 1.535	6.497 ± 2.878	15.100 ± 1.146	0.034 ± 0.015	0.135 ± 0.014	0.083 ± 0.033
X2278_C2_B6	B	CCR5	0.713 ± 0.403	5.480 ± 1.547	5.148 ± 2.220	0.021 ± 0.002	0.084 ± 0.016	0.231 ± 0.165
CE703010217_B6	C	CCR5	4.094 ± 1.399	0.631 ± 0.273	1.721 ± 0.112	0.047 ± 0.007	0.283 ± 0.063	0.188 ± 0.037
CE1176_A3	C	CCR5	4.501 ± 0.875	15.244 ± 4.760	42.505 ± 1.802	0.055 ± 0.007	0.326 ± 0.049	0.170 ± 0.096
HIV_25710-2.43	C	CCR5	2.917 ± 0.823	1.699 ± 0.826	4.437 ± 1.253	0.043 ± 0.016	0.104 ± 0.007	0.135 ± 0.045
X1632-S2-B10	G	CCR5	3.589 ± 1.206	13.855 ± 3.313	38.206 ± 4.066	0.055 ± 0.010	0.266 ± 0.094	9.826 ± 1.804
246_F3_C10_2	A/C	CCR5	3.822 ± 0.795	13.410 ± 5.506	26.853 ± 0.174	0.064 ± 0.019	0.683 ± 0.095	0.363 ± 0.077
CNE8	A/E	CCR5	7.022 ± 0.493	8.218 ± 3.908	19.323 ± 5.750	0.060 ± 0.005	0.295 ± 0.197	1.567 ± 0.409
CNE55	A/E	CCR5	2.748 ± 0.840	9.837 ± 3.116	12.136 ± 0.522	0.038 ± 0.020	0.169 ± 0.063	1.429 ± 0.274
CH119.10	B/C	CCR5	0.649 ± 0.202	9.144 ± 1.809	4.230 ± 0.170	0.020 ± 0.004	0.097 ± 0.021	0.118 ± 0.032
BJOX002000.03	B/C	CCR5	0.948 ± 0.130	2.628 ± 0.990	12.096 ± 0.685	0.035 ± 0.009	0.207 ± 0.023	0.211 ± 0.044
Mean			3.118	7.280	18.330	0.041	0.235	1.344

^*a*^ The experiments were performed in triplicate and repeated three times. Data are expressed as means ± standard deviations (SDs).

We further assessed the antiviral activities of 2P23, PRO 140, PRO140SC, and 2P23-PRO140SC along with m36.4-PRO140 using the second panel of pseudoviruses, which included 19 Envs selected from subtypes B, B′, C, and recombinant CRF01_AE and CRF01_BC forms that are currently circulating in China. As shown in Table 2, these five inhibitors had mean IC_50_ values of 2.424, 4.361, 12.892, 0.073, and 0.976 nM, respectively, indicating that 2P23-PRO140SC was 60-fold more potent than PRO 140, 177-fold more potent than PRO140SC, and 13-fold more potent than m36.4-PRO140. We also determined the inhibitory activities of 2P23, PRO140SC, 2P23-PRO140SC, and m36.4-PRO140 against HIV-1 Env-mediated cell-cell fusion by a DSP-based fusion assay. As shown in Table 3, PRO140SC could not inhibit the R4 virus NL4-3 and R5X4 virus R3A at a high concentration, leading to a mean IC_50_ greater than 398.83 nM, whereas 2P23, 2P23-PRO140SC and m36.4-PRO140 blocked the cell fusion efficiently, with mean IC_50_ values at 3.126, 0.346, and 11.486 nM, respectively.

**TABLE 2:** Inhibitory activities of PRO140-based inhibitors against divergent HIV-1 subtypes^*a*^

Pseudovirus	Subtype	Tropism	Mean IC_50_ ± SD (nM)				
			2P23	PRO 140	PRO140SC	2P23-PRO140SC	m36.4-PRO140
PVO	B	CCR5	6.673 ± 1.869	9.652 ± 3.825	12.680 ± 2.005	0.037 ± 0.009	0.857 ± 0.335
SC422661.8	B	CCR5	1.327 ± 0.202	6.086 ± 1.449	21.019 ± 1.991	0.022 ± 0.005	1.317 ± 0.502
JRFL	B	CCR5	8.436 ± 0.560	5.052 ± 0.199	20.011 ± 2.174	0.064 ± 0.048	0.067 ± 0.008
SF162	B	CCR5	4.011 ± 0.604	1.149 ± 0.376	2.348 ± 0.516	0.057 ± 0.018	0.558 ± 0.158
CNE4	B′	CCR5	2.424 ± 0.531	3.052 ± 0.946	6.562 ± 2.233	0.124 ± 0.034	0.230 ± 0.127
CNE6	B′	CCR5	1.182 ± 0.264	2.434 ± 0.456	4.355 ± 1.499	0.041 ± 0.009	0.132 ± 0.076
CNE9	B′	CCR5	0.998 ± 0.090	3.737 ± 1.177	6.189 ± 3.038	0.049 ± 0.010	0.250 ± 0.077
CNE11	B′	CCR5	3.231 ± 0.145	4.361 ± 1.411	11.328 ± 2.680	0.233 ± 0.067	0.242 ± 0.133
CNE14	B′	CCR5	3.270 ± 0.257	3.523 ± 1.219	2.970 ± 0.467	0.184 ± 0.021	0.180 ± 0.079
CNE57	B′	CCR5	1.041 ± 0.033	0.342 ± 0.053	0.764 ± 0.278	0.034 ± 0.011	0.086 ± 0.004
43–22	B′	CCR5	2.664 ± 0.155	1.642 ± 0.642	3.050 ± 0.562	0.027 ± 0.005	2.601 ± 0.298
B01	B′	CCR5	1.203 ± 0.123	5.260 ± 1.861	12.153 ± 6.646	0.082 ± 0.012	0.793 ± 0.189
CAP45.2.00.G3	C	CCR5	3.148 ± 0.312	5.099 ± 0.957	5.452 ± 0.746	0.054 ± 0.027	2.313 ± 2.098
Du156	C	CCR5	1.364 ± 0.297	11.918 ± 3.844	29.490 ± 9.158	0.029 ± 0.007	0.696 ± 0.084
AE03	A/E	CCR5	1.888 ± 0.088	1.847 ± 0.509	4.635 ± 0.279	0.038 ± 0.008	4.613 ± 1.287
CH64.20	B/C	CCR5	0.380 ± 0.009	10.567 ± 3.302	22.698 ± 6.418	0.035 ± 0.014	0.629 ± 0.247
CH70.1	B/C	CCR5	6.752 ± 1.637	2.457 ± 0.746	3.102 ± 0.440	0.075 ± 0.013	0.897 ± 0.283
CH110.2	B/C	CCR5	1.683 ± 0.267	13.329 ± 2.703	51.716 ± 12.863	0.049 ± 0.016	0.546 ± 0.055
CH120.6	B/C	CCR5	3.538 ± 0.305	9.829 ± 3.163	24.427 ± 16.281	0.152 ± 0.063	1.533 ± 0.675
Mean			2.424	4.361	12.892	0.073	0.976

^*a*^ The experiments were performed in triplicate and repeated three times. Data are expressed as means ± standard deviations (SDs).

**TABLE 3:** Inhibitory activities of PRO140-based inhibitors against HIV-1 Env-mediated cell fusion^*a*^

HIV-1 Env	Subtype	Tropism	Mean IC_50_ ± SD (nM)			
			2P23	PRO140SC	2P23-PRO140SC	m36.4-PRO140
NL4-3	B	CXCR4	0.214 ± 0.031	>1,858.529	0.091 ± 0.032	3.736 ± 0.962
R3A	B	R5/X4	5.857 ± 1.161	>371.706	0.366 ± 0.121	6.232 ± 1.875
CNE11	B′	CCR5	3.305 ± 0.078	22.136 ± 7.936	0.475 ± 0.266	4.921 ± 1.434
CAP210.2.00.E8	C	CCR5	1.015 ± 0.445	29.538 ± 6.692	0.111 ± 0.041	6.509 ± 2.852
CNE8	A/E	CCR5	5.720 ± 0.226	85.038 ± 16.275	0.503 ± 0.193	24.613 ± 8.878
CH70.1	B/C	CCR5	2.645 ± 0.078	26.034 ± 3.913	0.529 ± 0.156	22.906 ± 5.044
Mean			3.126	>398.83	0.346	11.486

^*a*^ The experiments were performed in triplicate and repeated three times. Data are expressed as means ± standard deviations (SDs).

**Fig 3:**
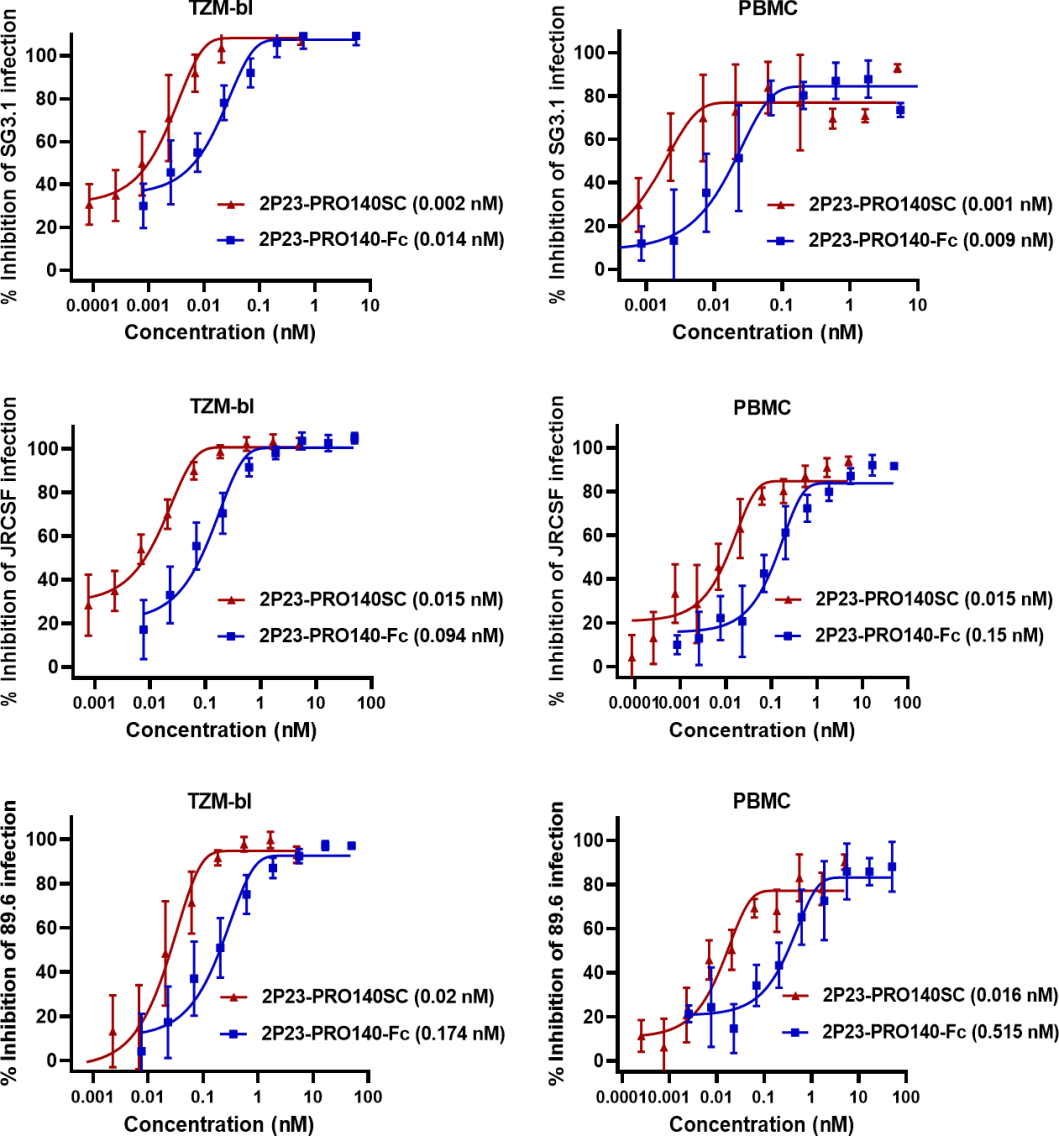
Inhibitory activities of 2P23-PRO140SC and 2P23-PRO140-Fc on replication-competent HIV-1 isolates. The activities of 2P23-PRO140SC and 2P23-PRO140-Fc in inhibiting CXCR4-tropic virus SG.1, CCR5-tropic virus JRCSF, and dual-tropic virus 89.6 were determined in TZM-bl cells (left panel) and human PBMC (right panel), respectively. The assays were performed in triplicate and repeated three times, and data are expressed as mean ± SD.

Furthermore, the anti-HIV activities of 2P23-PRO140SC and 2P23-PRO140-Fc were verified with three replication-competent HIV-1 viruses, including X4 virus SG3.1, R5 virus JRCSF, and R5/X4 virus 89.6 (Fig. 3). In the inhibition of virus infection on TZM-b1 cells, 2P23-PRO140SC and 2P23-PRO140-Fc inhibited SG3.1 with IC_50_ of 0.002 and 0.014 nM, respectively; inhibited JRCSF with IC_50_ of 0.015 and 0.094 nM, respectively; and inhibited 89.6 with IC_50_ of 0.02 and 0.174 nM, respectively. In the inhibition of virus infection on human peripheral blood mononuclear cell (PBMC) culture, 2P23-PRO140SC inhibited the three viruses with IC_50_ of 0.001, 0.015, and 0.016 nM, respectively, whereas 2P23-PRO140-Fc behaved with IC_50_ of 0.009, 0.15, and 0.515 nM, respectively. Taken together, our results demonstrated that PRO 140 and 2P23-based bispecific inhibitors possess very potent and broad-spectrum anti-HIV activities.

### 2P23-PRO140SC is a highly potent inhibitor of 2P23 and T20-resistant HIV-1 mutants

We previously reported a panel of 2P23 inhibitor-resistant HIV-1 mutants that carry the single, double, or triple mutations in the gp41 target site. Herein, the corresponding mutant pseudoviruses were prepared, and single-cycle infection assays were performed to characterize the inhibitory activities of 2P23-PRO140SC along with 2P23, PRO140SC, and m36.4-PRO140 for comparison. In agreement with the previous data, the mutant viruses conferred various degrees of resistance to the inhibition of 2P23 with a mean IC_50_ at 21.132 nM and a mean resistance fold change (FC) of 27.805-fold; however, 2P23-PRO140SC could potently inhibit the panel of the viruses with a mean IC_50_ at 0.025 nM and a mean FC at 2.52-fold (Table 4). Specifically, the L57R and L57R/E136G mutants displayed the FC values of 58.688 and 182.233 to 2P23, but they were not resistant to 2P23-PRO140SC. It was anticipated that PRO140SC had no inhibitory activity in inhibiting the X4 virus (NL4-3)-based mutants; but unanticipatedly, most mutants exhibited mild resistance to m36.4-PRO140, which targets gp120 and CCR5 rather than gp41, leading to a mean IC_50_ of 1.314 nM with a mean FC value at 4.043.

**TABLE 4:** Inhibitory activities of PRO140-based inhibitors and 2P23 peptide against 2P23- and T20-resistant HIV-1_NL4-3_ mutants^*a*^

Pseudovirus	2P23		PRO140SC	2P23-PRO140SC		m36.4-PRO140	
	IC_50_ ± SD (nM)	Fold change	IC_50_ ± SD (nM)	IC_50_ ± SD (nM)	Fold change	IC_50_ ± SD (nM)	Fold change
Wild type	0.764 ± 0.280	1	>1,858.529	0.010 ± 0.001	1	0.325 ± 0.058	1
2P23 resistants							
E49A	3.141 ± 1.109	4.133	>1,858.529	0.010 ± 0.002	1.000	1.556 ± 0.799	4.788
E49K	3.089 ± 1.120	4.064	>1,858.529	0.012 ± 0.003	1.200	1.530 ± 0.594	4.708
Q52R	3.428 ± 1.212	4.511	>1,858.529	0.009 ± 0.004	0.900	2.118 ± 1.319	6.517
L57R	44.603 ± 11.762	58.688	>1,858.529	0.004 ± 0.001	0.400	0.657 ± 0.185	2.022
E136G	5.442 ± 1.119	7.161	>1,858.529	0.053 ± 0.010	5.300	0.062 ± 0.002	0.191
N43K/E49A	2.561 ± 0.093	3.370	>1,858.529	0.013 ± 0.002	1.300	1.716 ± 0.698	5.280
E49K/N126K	3.412 ± 1.031	4.489	>1,858.529	0.046 ± 0.014	4.600	2.948 ± 0.167	9.071
L57R/E136G	138.497 ± 14.206	182.233	>1,858.529	0.010 ± 0.003	1.000	0.943 ± 0.551	2.902
Q39R/N43K/N126K	1.898 ± 0.139	2.497	>1,858.529	0.061 ± 0.019	6.100	0.500 ± 0.323	1.538
N43K/E49A/N126K	5.246 ± 1.837	6.903	>1,858.529	0.034 ± 0.017	3.400	1.110 ± 0.513	3.415
Mean	21.132	27.805	>1,858.529	0.025	2.520	1.314	4.043
T-20 resistants							
I37T	0.637 ± 0.089	0.838	>1,858.529	0.025 ± 0.004	2.500	0.187 ± 0.255	0.575
V38A	0.619 ± 0.141	0.814	>1,858.529	0.016 ± 0.003	1.600	0.782 ± 0.403	2.406
V38M	0.686 ± 0.076	0.903	>1,858.529	0.023 ± 0.002	2.300	1.119 ± 0.581	3.443
Q40H	0.689 ± 0.166	0.907	>1,858.529	0.014 ± 0.005	1.400	0.148 ± 0.089	0.455
N43K	0.560 ± 0.041	0.737	>1,858.529	0.015 ± 0.002	1.500	0.564 ± 0.258	1.735
D36S/V38M	0.940 ± 0.046	1.237	>1,858.529	0.049 ± 0.014	4.900	0.198 ± 0.040	0.609
V38A/N42T	0.412 ± 0.146	0.542	>1,858.529	0.010 ± 0.001	1.000	0.066 ± 0.023	0.203
I37T/N43K	0.838 ± 0.095	1.103	>1,858.529	0.021 ± 0.004	2.100	0.057 ± 0.036	0.175
Mean	0.673	0.885	>1,858.529	0.022	2.163	0.390	1.200

^*a*^ The experiments were performed in triplicate and repeated three times. Data are expressed as means ± standard deviations (SDs).

We also evaluated the inhibitory activities of these four inhibitors against a panel of T20-resistant mutants. As shown in Table 4, 2P23, 2P23-PRO140SC, and m36.4-PRO140 inhibited this panel of mutant viruses very efficiently, exhibiting the mean IC_50_ values at 0.673, 0.022, and 0.39 nM, respectively. It was interesting to find that the D36S/V38M mutant displayed mild-level resistance to 2P23-PRO140SC (FC = 4.9), whereas it was sensitive to 2P23 and m36.4-PRO140. Combined, these results suggest that 2P23-PRO140SC is a highly potent inhibitor of HIV-1 mutants that are resistant to 2P23 or T20.

### 2P23-PRO140SC has synergistic anti-HIV effects with 2P23-iMab and m36.4-PRO140

**Fig 4:**
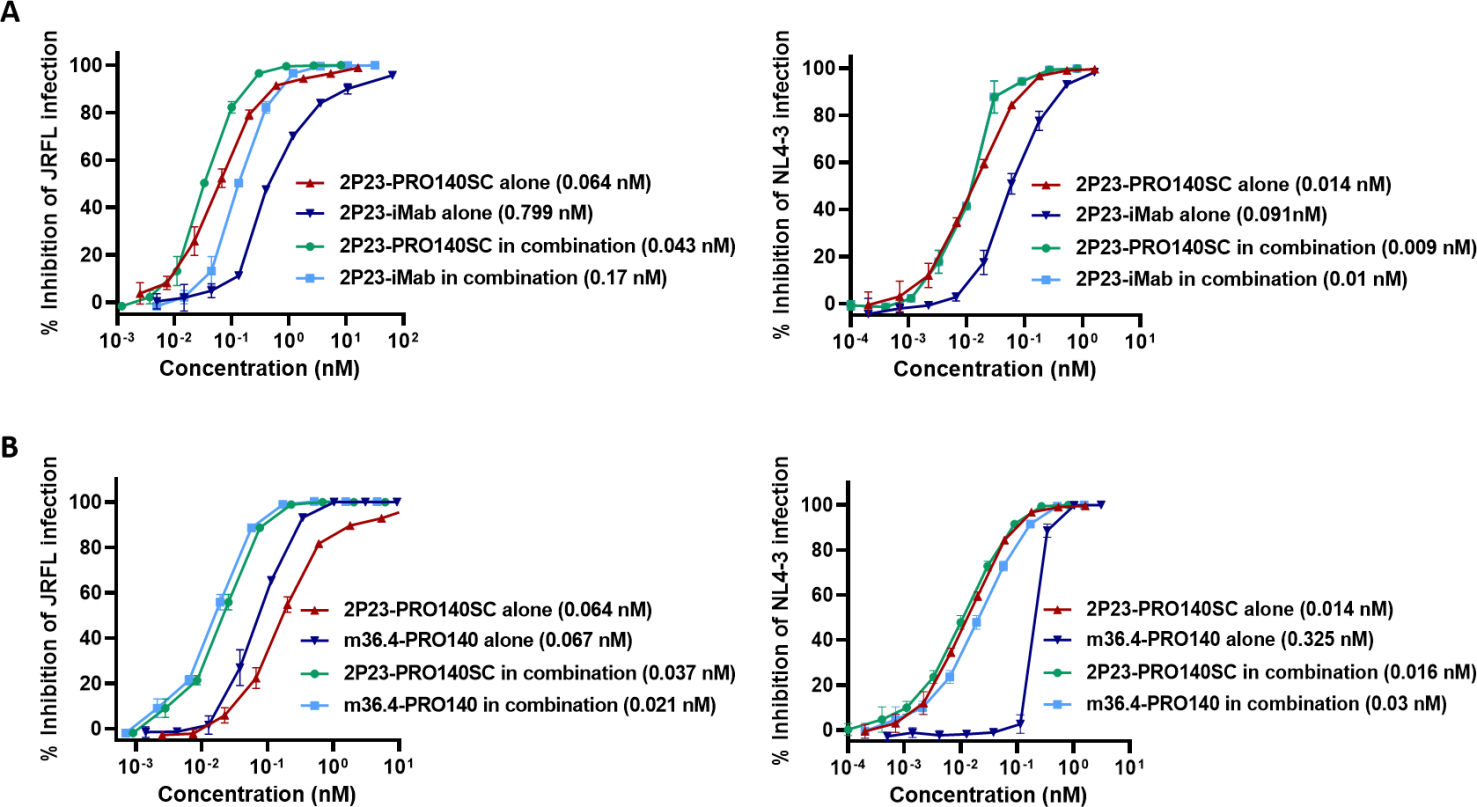
Synergistic effects of 2P23-PRO140SC with bispecific inhibitors 2P23-iMab and m36.4-PRO140. (**A**) Inhibitory activities of 2P23-PRO140SC and 2P23-iMab alone or in combination against HIV-1 JRFL (left panel) and NL4-3 (right panel) pseudoviruses. (**B**) Inhibitory activities of 2P23-PRO140SC and m36.4-PRO140 alone or in combination against HIV-1 JRFL (left panel) and NL4-3 (right panel) pseudoviruses. The assays were performed in triplicate and repeated three times, and mean IC_50_ values are shown in parentheses.

We previously designed the fusion protein 2P23-iMab that exerts bifunctional inhibition through CD4-targeting antibody ibalizumab (27). Considering the different mechanisms of the two anchoring antibodies, we were interested in characterizing whether 2P23-PRO140SC and 2P23-iMab had a synergistic anti-HIV activity. For this, two inhibitors were mixed at an indicated molar ratio, and their inhibitory activities in combination or alone were determined by the pseudovirus-based infection assay. As shown in Fig. 4A, the combination of 2P23-PRO140SC and 2P23-iMab brought about a moderate synergistic interaction against the infections of JRFL and NL4-3, exhibiting the CI values of 0.813 and 0.804, respectively. In the inhibition of JRFL, a 1.5-fold reduction for 2P23-PRO140SC and a 4.7-fold reduction for 2P23-iMab in IC_50_ values were observed when they were in combination. In the inhibition of NL4-3, the combined inhibitors showed 1.6-fold and 9.1-fold decreased IC_50_ values.

We also had a curiosity to know whether 2P23-PRO140SC had a synergy with m36.4-PRO140 even though both the inhibitors work through CCR5 anchoring. As shown in Fig. 4B, a slight synergistic effect was also found in the inhibition of JRFL infection with a CI value of 0.889; but surprisingly, the combination of 2P23-PRO140SC and m36.4-PRO140 had a slight antagonistic effect in the inhibition of NL4-3 infection, as indicated by the CI value of 1.232.

### The *in vitro* cytotoxicity and stability of 2P23-PRO140SC and 2P23-PRO140-Fc

**Fig 5:**
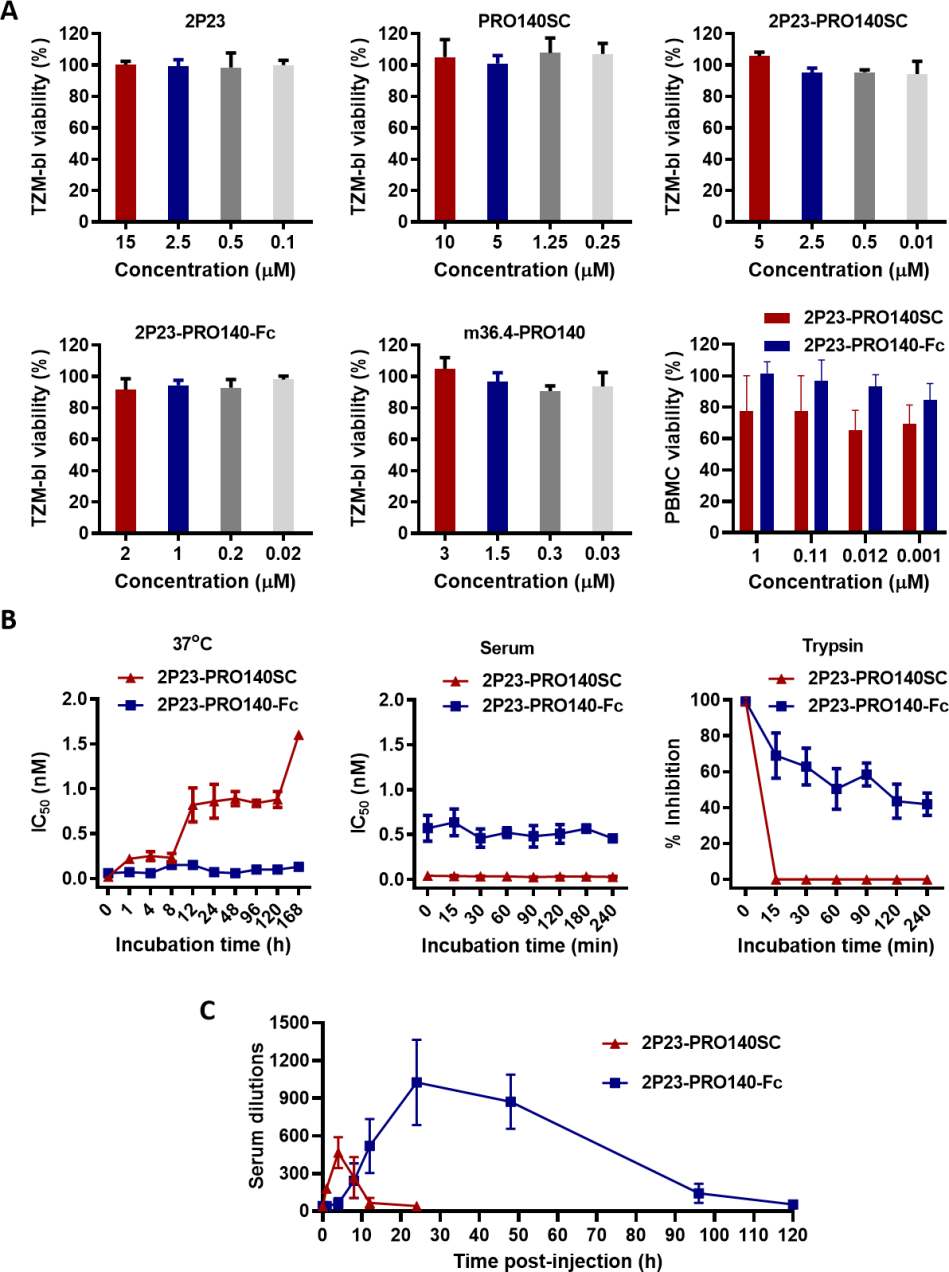
The *in vitro* cytotoxicity, metabolic stability, and *in vivo* anti-HIV activity of 2P23-PRO140SC and 2P23-PRO140-Fc. (**A**) While the cytotoxicity of most PRO 140-based inhibitors along with 2P23 peptide on TZM-bl cells was determined, the cytotoxicity of 2P23-PRO140SC and 2P23-PRO140-Fc on human PBMC was also determined, using a method of CCK-8 kit. The experiments were performed in triplicate and repeated three times. Data are expressed as mean ± SD. (**B**) Metabolic stabilities of 2P23-PRO140SC and 2P23-PRO140-Fc when stored at 37°C, incubated in rat serum or digested with trypsin. The anti-HIV activities of treated fusion proteins were measured by HIV-1 NL4-3-based single-cycle infection assay. (**C**) The *in vivo* anti-HIV activity of 2P23-PRO140SC and 2P23-PRO140-Fc. The fusion protein proteins were subcutaneously administered to rats at a dose of 3 mg/kg, and serum samples were collected over time for the measurement of anti-HIV activity by the HIV-1 NL4-3-based single-cycle infection assay; serum dilutions required for 50% inhibition of virus infection were calculated.

The *in vitro* cytotoxicity of 2P23-PRO140SC and 2P23-PRO140-Fc together with 2P23, PRO140SC, and m36.4-PRO140 was examined by a standard CCK8 assay. As shown in Fig. 5A, none of the inhibitors showed appreciable cytotoxicity on TZM-bl cells at a high concentration, indicating a relatively high therapeutic index (IC_50_/CC_50_). When human PBMC was applied, no obvious cytotoxicity was observed either, but 2P23-PRO140SC did cause certain degrees of decrease in cell viability with the relatively large variations.

To characterize the metabolic stability of 2P23-PRO140SC and 2P23-PRO140-Fc, the protein solutions were treated with different experimental conditions, and their anti-HIV activities were measured with the HIV-1 NL4-3 pseudovirus-based single-cycle infection assay. First, the protein solutions were stored at 37°C for 1 week, and the samples were collected at different time points. As shown in Fig. 5B, the antiviral potency of 2P23-PRO140SC markedly reduced over time, whereas 2P23-PRO140-Fc tolerated the temperature treatment satisfactorily. Second, the protein solutions were subjected to a rat serum and incubated at 37°C for 4 h. As shown, both proteins were highly resistant to the serum treatment over time, with the IC_50_ values nearly unchanged. Third, when the proteins were digested with trypsin, their antiviral activities were severely impaired. Collectively, these results highlight the biophysical properties of the recombinant protein inhibitors.

### The *in vivo* antiviral activities of 2P23-PRO140SC and 2P23-PRO140-Fc

We previously evaluated the *in vivo* antiviral activities of multiple HIV fusion inhibitors with animal models without animal infection facilities (20, 26, 29, 30). Herein, we explored the *in vivo* anti-HIV performance of 2P23-PRO140SC and 2P23-PRO140-Fc inhibitors in an SD rat model. Ten SD rats were divided into two groups of five rats each and administered the fusion proteins at a dose of 3 mg/kg by subcutaneous injection; blood was collected at different time points for measuring the anti-HIV activity by the single-cycle infection assay. As shown in Fig. 5C, 2P23-PRO140SC rapidly reached a high serum anti-HIV activity at 1 h post-injection with a mean serum dilution of 181-fold that inhibited 50% of virus infection, and it exhibited a mean serum peak level at 4 h post-injection with a serum dilution at 466-fold. Significantly, the sera of 2P23-PRO140-Fc-treated rats exhibited a mean dilution of 243-fold after injection 8 h and a mean peak level from 24 to 48 h post-injection with serum dilutions ranging from 871-fold to 1,025-fold. Even after injection 96 h, 2P23-PRO140-Fc still retained the inhibitory activity with a mean serum dilution of 143-fold, suggesting that 2P23-PRO140-Fc possessed enhanced *in vivo* antiviral activity with a prolonged half-life.

## DISCUSSION

The primary goal of this study is to develop a novel anti-HIV drug candidate that possesses the “two-birds-one-stone” effect via a dual-target design strategy. To achieve this aim, we genetically linked the fusion-inhibitory peptide 2P23 and the scFv of PRO 140 antibody, generating two tandem fusion proteins 2P23-PRO140SC and 2P23-PRO140-Fc that enabled the simultaneous targeting of the cell coreceptor CCR5 and viral fusion protein gp41. As demonstrated, both the fusion protein inhibitors exhibited dramatically improved anti-HIV activities relative to the template inhibitors (2P23 and PRO140SC), not only against CCR5-tropic viruses but also CXCR4-tropic HIV-1 viruses. They were also highly active in inhibiting 2P23- and T20-resistant HIV-1 variants that possess amino acid mutations on the target sites of gp41. We also found that 2P23-PRO140SC displayed synergistic effects with the previously designed bispecific inhibitors 2P23-iMab and m36.4-PRO140, which potently blocked HIV-1 infection through CD4-anchoring or CCR5 anchoring. Moreover, both 2P23-PRO140SC and 2P23-PRO140-Fc exhibited very low *in vitro* cytotoxicity and high *ex vivo* anti-HIV activity in administered rats, with 2P23-PRO140-Fc being much better than 2P23-PRO140SC in terms of their inhibition potencies and long-lasting effects. In one-sentence summary, our studies have not only provided bispecific HIV-1 inhibitors for potential clinical development but also offered new tools for studying the mechanisms of HIV-1 entry and its inhibition.

CCR5, a C-C chemokine receptor 5, is a G protein-coupled receptor involved in cell signaling and migration, being mostly expressed on the surface of lymphocytes, macrophages, dendritic cells, and natural killer cells (6, 31). The primary CCR5 ligands are chemokines CCL3 (MIP-1a), CCL4 (MIP-1b), and CCL5 (RANTES), which are expressed in sites of inflammation to recruit CCR5^+^ immune cells; thus, CCR5 plays a critical role in inflammatory responses and other immune functions. As a primary cell coreceptor for HIV-1, CCR5 dictates the cell tropism and has been considered an ideal target for developing therapeutic strategies (5, 10). After MVC, PRO 140 antibody is one of the most promising anti-HIV drug candidates targeting CCR5 (17, 32). However, the inability of PRO 140 on CXCR4-tropic and dual-tropic viruses is one of its shortcomings, which has significantly limited its clinical application. The previous and ongoing clinical studies of PRO 140 have demonstrated its promising efficacy against CCR5-tropic viruses, including as a maintenance monotherapy for virologically suppressed patients and for treatment-experienced patients with multidrug-resistant virus (16, 17, 32, 33). To date, however, only a small number of study participants have achieved durable viral suppression with PRO 140 monotherapy, suggesting that most patients require combinations with other antiretroviral drugs (32). In this work, the rational design of the tandem fusion proteins by conjugating PRO 140 with a short peptide has finely overcome the problems above, as the bispecific inhibitors can simultaneously block the binding of the virus to CCR5 and gp41-mediated cell fusion, thereby being the extraordinarily potent inhibitors of HIV-1 isolates independent of their cell tropism. As one of the antiviral mechanisms, the CCR5 anchoring can greatly elevate the local concentration of the fusion-inhibitory peptide 2P23 at the virus-cell fusion site, thus blocking HIV-1 entry more efficiently.

Drug resistance is one of the most concerning issues in the HIV-1 treatment field, and cART is required for the effective suppression of viral replication. Indeed, the rapid emergence and spread of drug-resistant HIV-1 isolates has led to an increasing number of patients who have not responded to previous treatment (34, 35). Different from those drugs directed to the viral proteins, it is difficult for HIV-1 to develop resistance against the host proteins. Indeed, the clinical studies demonstrated that no development of R5 viral resistance was observed following treatment with single or multiple doses of PRO 140, indicating no adaptation of the virus to use CCR5 in the presence of the drug (16, 18). Nonetheless, concerns remain over the development of HIV-1 resistance to PRO 140 due to a switched tropism after long-term drug pressure (9, 32). As described in the Introduction section, the fusion inhibitor 2P23 peptide was rationally designed with a mixed sequence of HIV-1, HIV-2, and SIV, and its N-terminal contained an M-T hook structure to target the highly conserved pocket site of gp41, making the inhibitor with a very potent, broad-spectrum antiviral activity, as well as a high genetic barrier to inducing drug resistance (25). In comparison, 2P23 exhibited markedly improved activities over enfuvirtide (T-20) in inhibiting divergent HIV-1, HIV-2, and SIV isolates, and it also maintained its high potency against enfuvirtide-resistant mutants (25, 26). In this study, the fusion protein inhibitors displayed very high potencies against divergent HIV-1 subtypes and fusion inhibitor-resistant mutants; thus, it is conceivable that adding 2P23 to PRO 140 would enable a further increased genetic barrier for virus resistance. In our future works, we will study the resistance profiles of 2P23-PRO140SC or 2P23-PRO140-Fc by selecting drug-resistant HIV-1 mutants and characterizing the mechanism underlying the resistance phenotypes.

Due to the persistence of viral reservoirs, the goal of current HIV treatment is to achieve a functional cure. Recent studies have demonstrated that allogeneic hematopoietic stem cell transplantation (HSCT) with homozygous CCR5 delta32 mutations (CCR5*Δ32/Δ32*) is a powerful strategy, and several patients have been functionally cured, including the “Berlin patient” (36), the “London patient” (37), and the “Dusseldorf patient” (38). Due to the low number of homozygous CCR5Δ32 donors in the population as well as the concerns over the safety of treatment, HSCT is not a viable option for most HIV-infected patients; therefore, alternative strategies for HIV-1 functional cure targeting CCR5 have been extensively explored (10, 39, 40). There are intensive works on the disruption of CCR5 expression by gene-editing tools, such as CRISPR/Cas9, ZFN, and TALEN-based technologies; however, the clinical efficacies of CCR5 editing are limited (1, 41–43). Although 2P23-PRO140SC exhibited highly potent *in vitro* anti-HIV activity, its *in vivo* potency might be significantly restricted by the pharmacokinetics with a short half-life, as indicated by the *ex vivo* data in rats. The engineering of 2P23-PRO140SC with IgG4 Fc greatly improved the case, but they still need continuous optimizations to satisfy the long-term treatment in clinical settings. The HSCT-based therapy also informs that a functional cure can be achieved through the infusion of HIV-resistant cells for gene therapy. In this track, we have modified CD4^+^ target cells with 2P23 peptide, antibody, or bispecific fusion proteins through glycosylphosphatidylinositol anchoring, which can render the cells fully resistant to HIV-1 infection (44–47). Therefore, we propose that 2P23-PRO140SC and/or 2P23-PRO140-Fc can be used for CCR5-targeting gene therapy by delivering them through a genetic vector, such as lentiviral vector and adeno-associated virus vector. Toward this goal, we have an ongoing research project to generate HIV-resistant cells that can secrete 2P23-PRO140SC molecules.

Besides the works mentioned above, several other studies are also needed to address the limitations of the present results. First, the mechanism underlying the potent anti-HIV activity of the fusion proteins should be further characterized; for example, how they regulate the spatiotemporal relationship to overcome the steric hindrance during the virus-cell fusion process. Second, it is very important to investigate the *in vivo* pharmacokinetics, safety, and therapeutic efficacy of the fusion proteins in animal models prior to their further development. Although we show that the dose-dependent cell membrane anchoring of the inhibitors does not affect the expression level of CCR5 receptor, these are likely to be far higher on TZM-bl cells than on primary CD4^+^ T cells, their presumed *in vivo* target. Third, we are interested in studying whether the 2P23 peptide can be directly conjugated to a prototype antibody of PRO 140 that maintains the antiviral potency of scFv-based fusion proteins but with enhanced *in vivo* stability.

## MATERIALS AND METHODS

### Reagents and cells

Plasmids encoding the “global panel” HIV-1 Envs (subtypes A, B, C, G, A/C, A/E, and B/C) and TZM-bl cells were obtained through the AIDS Reagent Program, Division of AIDS, NIAID, NIH. Plasmids encoding the Envs derived from subtype B′ (CNE4, CNE6, CNE9, CNE11, CNE14, and CNE57), CRF01_AE (CNE107), and CRF07_BC (CNE49) were kindly gifted by Linqi Zhang at the Tsinghua University, Beijing, China. Three plasmids encoding subtype B′ (B01 and 43-22) and CRF01_AE (AE03) Envs were kindly provided by Youchun Wang at the National Institute for the Control of Pharmaceutical and Biological Products, Beijing, China. Four plasmids encoding CRF07_BC Envs (CH64.20, CH70.1, CH110.2, and CH120.6) were kindly gifted by Yiming Shao at the Chinese Center for Disease Control and Prevention, Beijing, China. A plasmid encoding DSP_1-7_ and 293 FT cells stably expressing CXCR4/CCR5/DSP_8-11_ were kindly provided by Zene Matsuda at the University of Tokyo, Tokyo, Japan. HEK293T cells were purchased from the American Type Culture Collection (ATCC) (Rockville, MD, USA). The cells were cultured in Dulbecco’s minimal essential medium (DMEM) containing 10% fetal bovine serum (FBS), 100 U/mL of penicillin-streptomycin, 2 mM l-glutamine, and 1 mM sodium pyruvate in a 5% CO_2_ incubator at 37°C. 2P23 peptide (EMTWEEWEKKVEELEKKIEELLK) was synthesized and used in our previous studies (25, 27). A mouse mAbs (5F7) was isolated in our laboratory, and its specificity to 2P23 peptide was characterized by enzyme-linked immunosorbent assay (ELISA) and western blot analysis assay (Fig. S3).

### Construction, expression, and purification of PRO 140-based inhibitors

The scFv of PRO 140 was designated PRO140SC. A bispecific inhibitor targeting CCR5 and gp41, designated 2P23-PRO140SC, was genetically constructed by fusing 2P23 peptide to the N-terminal of PRO140SC via a GGGGS linker sequence of three repeats. 2P23-PRO140-Fc was further engineered by fusing 2P23-PRO140SC to the N-terminal of IgG4 Fc that possessed M428L and N434S mutations. As a control, a previously reported bispecific inhibitor m36.4-PRO140 was also constructed by attaching m36.4 (a single-domain antibody) targeting the CD4-induced epitope on gp120 to the N-terminal of the PRO140SC via the GGGGS linker as described previously (48). The constructs contained an N-terminal secretory signal peptide of IgG3 leader to enhance the protein release and a C-terminal His tag for detection and purification.

HEK293T cell lines that stably express recombinant PRO140SC, 2P23-PRO140SC, and 2P23-PRO140-Fc proteins were generated with a lentiviral vector as described previously (27). Briefly, fusion genes encoding the constructs and green fluorescent protein (GFP) linked via a furin-GSG-T2A peptide signal were synthesized (SinoGenoMax, Beijing, China) and then cloned into a hPGK-driven self-inactivating lentiviral transfer vector pRRLsin.PPT.hPGK.WPRE between the BamHI and SalI sites. To package lentiviral particles, 1.5 × 10^7^ HEK293T cells were seeded in P-150 dishes in 25 mL of complete DMEM medium and cultured at 37°C overnight, and then cotransfected with the lentiviral transfer vector encoding the fusion genes (50 µg), packaging plasmid delta8.9 encoding Gag/Pol/Rev (18.75 µg), and the plasmid encoding vesicular stomatitis virus G envelope (VSV-G) (7.5 µg) by a linear polyethyleneimine (PEI) transfection reagent. Culture supernatant was discarded and replaced with fresh complete DMEM containing 10% FBS at 20 h posttransfection and cultured for another 24 h. The supernatants containing virus particles were harvested, centrifuged at 4,000 rpm for 15 min and filtered through a 0.45 µm filter. After concentrating by ultracentrifugation at 25,000 rpm for 2 h, precipitated pellets were resuspended in complete DMEM with 10% FBS and stored in aliquots at −80°C. The viral titers were determined with HEK293T cells by monitoring the expression of GFP with a FACSCanto II instrument (Becton-Dickinson, Mountain View, CA, USA) and were expressed as transducing units (TU) per milliliter. Next, a total of 1 × 10^5^ HEK293T cells were seeded in a 24-well plate and cultured at 37°C overnight; the cells were added with 1 × 10^6^ TU of recombinant lentiviruses and supplemented with 8 µg/mL polybrene (Sigma, St. Louis, MO, USA). After culturing for 24 h, transduced cells were extensively washed and cultured in complete DMEM. HEK293T cells expressing the transgenes were sorted and collected by GFP expression. After incubation for 48 h, recombinant proteins were purified from culture supernatants using affinity chromatography. Following elution from a nickel affinity column, protein buffer was exchanged into phosphate-buffered saline (PBS, pH 7.4) using Amicon Ultra-4 centrifugal filter units (Millipore, Billerica, MA, USA).

To express a full-length PRO 140 antibody, the genes encoding its heavy chain and light chain were respectively synthesized and inserted into a cytomegalovirus-driven expression vector pcDNA3.4 at the HindIII/EcoRI or HindIII/ApaI restriction sites. The heavy chain contained a His tag at the C-terminus for detection and purification. HEK293T cells were cotransfected with plasmids encoding the heavy and light chains by a linear PEI transfection reagent. After culturing 48 h, PRO 140 was purified from cell supernatants using protein A affinity chromatography, and elution buffer was exchanged into PBS (pH 7.4) using Amicon Ultra-4 centrifugal filter units as above.

### Sodium dodecyl sulfate-polyacrylamide gel electrophoresis (SDS-PAGE) and western blot analysis

The purity and molecular size of purified recombinant protein inhibitors were characterized by SDS-PAGE, in which protein samples were loaded onto a 10% SDS-PAGE separating gel with equal mass, and the gel was then stained with Coomassie brilliant blue. Western blot analysis assay was performed as described previously (27). Briefly, equal amounts of the proteins were separated by 10% SDS-PAGE gel and then transferred to a nitrocellulose membrane, followed by blocking with a 5% (wt/vol) solution of nonfat dry milk in Tris-buffered saline-Tween 20 (TBST, pH 7.4) for 1 h at room temperature. The membrane was incubated with a mouse anti-His tag antibody (Sigma Aldrich, St. Louis, MO, USA) at 1:3,000 dilution or anti-2P23 peptide antibody 5F7 (4 µg/mL) overnight at 4°C. After three washes with TBST, the membrane was incubated with IRDye 680RD-conjugated Goat anti-Mouse immunoglobulin G (IgG) antibody (Rockland, Philadelphia, PA, USA) at 1:20,000 dilution for 1 h at room temperature. Imaging was performed using the Odyssey infrared imaging system (LI-COR Biosciences, Lincoln, NE, USA).

### Flow cytometry analysis

The binding of inhibitors to cell membranes was determined by a flow cytometry assay as described previously (27). Briefly, a protein or 2P23 peptide was added to TZM-bl cells (1 × 10^6^) and incubated for 1 h at 4°C. After being washed twice with FACS buffer (PBS supplemented with 0.5% bovine serum albumin and 2 mM EDTA), the cells were incubated with a mouse anti-His tag antibody (Sigma Aldrich, St. Louis, MO, USA) at 1:200 dilution for 1 h at 4°C. The cells were then washed twice and incubated with an Alexa Fluor 488 rabbit anti-mouse IgG antibody (Invitrogen, Carlsbad, CA, USA) for 1 h at 4°C. Next, the cells were stained with APC-mouse anti-human CD195 (CCR5) antibody (BD Biosciences, Franklin Lakes, NJ, USA) for 1 h at 4°C. After being washed twice, the cells were resuspended by FACS buffer containing 4% formaldehyde, and FACS analysis was conducted with the FACSCanto II instrument. To determine the dose-dependent binding of the inhibitors, 100 µL of inhibitors diluted at graded concentrations was added to 1 × 10^6^ TZM-bl cells and incubated for 1 h at 4°C. After the cells were washed twice with FACS buffer, APC-mouse monoclonal to His tag (Abcam, Cambridge, UK) and PE-mouse anti-human CD195 antibody (BD Biosciences) were respectively added, and incubation was continued at 4°C for 30 min. After thorough washing with FACS buffer, stained cells were resuspended in 0.2 mL of FACS buffer and analyzed with a FACS Canto II instrument.

### Single-cycle infection assay

The antiviral activities of inhibitors against divergent HIV-1 subtypes and resistant mutants were determined by a pseudovirus-based single-cycle infection assay as described previously (27). Briefly, viral stocks were generated by cotransfecting HEK293T cells with a viral backbone plasmid p*SG3^∆env^* and an Env-encoding plasmid using a linear PEI transfection reagent. Cell supernatants containing pseudovirus were harvested 48 h after transfection and stored in aliquots at −80°C. 50% tissue culture infectious dose (TCID_50_) was measured in TZM-bl cells. A serially threefold diluted peptide or protein inhibitor was incubated with 100 TCID_50_ of viruses at 37°C for 1 h, and then the inhibitor-virus mixture was added to TZM-bl cells (10^4^/well in a 100 µL volume). After incubation at 37°C for 48 h, the cells were harvested and lysed in reporter lysis buffer, followed by the measurement of luciferase activity using luciferase assay reagents and a luminescence counter (Promega, Madison, WI, USA). The percentage of inhibition and 50% inhibitory concentration (IC_50_) of an inhibitor were calculated using GraphPad Prism 8 software (GraphPad Software Inc., San Diego, CA, USA).

The synergistic effects between the two inhibitors were also determined by the single-cycle infection assay, in which the inhibitors were tested individually or mixed at a fixed molar ratio based on their individual IC_50_ values against the HIV-1 pseudoviruses. Similarly, the inhibitors were serially diluted at threefold, 100 TCID_50_ of viruses, and 1 × 10^4^ cells/well of TZM-bl cells were used. The cooperative inhibition effects were analyzed by referring to the method of Chou and Talalay (49). A CI was calculated by using the CalcuSyn 2.0 program. A CI of <1 indicates synergism (<0.1, very strong synergism; 0.1–0.3, strong synergism; 0.3–0.7, synergism; 0.7–0.85, moderate synergism; and 0.85–0.9, slight synergism), a CI of 1 or close to 1 indicates additive effects, and a CI of >1 indicates antagonism.

### Cell-cell fusion assay

A dual-split-protein (DSP)-based cell-cell fusion assay was performed as described previously (27). Briefly, 1.5 × 10^4^ HEK293T cells (effector cells) were seeded in a 96-well plate and cultured at 37°C overnight. The cells were then cotransfected with a mixture of an Env-expressing plasmid and a DSP_1–7_-expressing plasmid and incubated at 37°C for 24 h. Next, 293 FT cells that stably express CXCR4/CCR5 and DSP_8-11_ (target cells) were resuspended with the addition of ~17 µg/mL EnduRen live cell substrate (Promega) and incubated at 37°C for 30 min. Then, the target cells (3 × 10^4^/well) were added to the effector cell wells with or without diluted inhibitors at graded concentrations. The cell mixtures were spun down to facilitate cell-cell contact and incubated for 2 h at 37°C. Luciferase activity was measured with luciferase assay reagents, and IC_50_ values of the inhibitors were calculated as described above.

### Inhibition of replication-competent HIV viruses

The inhibitory activities of inhibitors on three replication-competent HIV isolates (SG3.1, JRCSF, and 89.6) were performed as described previously (20). Viral stocks were prepared by transfecting viral molecular clones into HEK293T cells, and TCID_50_ was measured in TZM-bl cells. For the inhibition of TZM-bl cells, an inhibitor was prepared in threefold dilutions, mixed with 100 TCID_50_ of viruses, and then incubated for 1 h at room temperature. Next, 100 µL of the inhibitor-virus mixture was added to 1 × 10^4^ TZM-bl cells and incubated for 48 h at 37°C. The luciferase activity was determined using luciferase assay reagents and a luminescence counter as above. For the inhibition of human PBMCs, 4 × 10^5^ human PBMCs were first stimulated with 5 µg/mL phytohemagglutinin (PHA) and 50 U/mL interleukin-2 (IL-2) for 3 days. A serially diluted inhibitor was incubated with 200 TCID_50_ of viruses and then added to 4 × 10^5^ human PBMCs, followed by incubation at 37°C for 4 h. The inhibitor-virus mixture was replaced with fresh culture medium and incubated for an additional 3 days at 37°C. Viral p24 antigen in the culture supernatant was measured by an ELISA kit. The percent inhibition and IC_50_ values were calculated using GraphPad Prism 8 software.

### Cytotoxicity of bispecific inhibitors

Cytotoxicities of bispecific inhibitors on TZM-bl cells or human PBMCs were measured using cell counting kit-8 (CCK-8) (Abbkine, Wuhan, China). Briefly, 50 µL of inhibitors diluted at graded concentrations were added to cells, which were seeded with 1 × 10^4^ cells/well (TZM-bl) or 4 × 10^5^ cells/well (human PBMC) on a 96-well tissue culture plate. After incubation at 37°C for 48 h (TZM-bl) or 72 h (human PBMC), 20 µL of CCK-8 solution reagent was added into each well and incubated for 2 h at 37°C. The absorbance was measured at 450 nm using a Multiscan MK3 microplate reader (Thermo Fisher Scientific, Waltham, MA, USA), and the cell viability (percentage) was calculated.

### Metabolic stability of bispecific inhibitors

To detect the *in vitro* metabolic stability of bispecific inhibitors, two fusion protein solutions were stored at 37°C for 0, 1, 4, 8, 12, 24, 48, 120, and 168 h; or incubated at 37°C in PBS containing trypsin (Sigma-Aldrich, St. Louis, MO, USA) with a trypsin/protein mass ratio of 1:20 for 0, 15, 30, 60, 90, 120, and 240 min; or incubated at 37°C with the serum from a 6-week SD rat with a serum/protein volume ratio of 1:20 for 0 h, 1 h, 4 h, 8 h, 12 h, 24 h, 2 days, 5 days, and 7 days). All samples were collected from different time points and stored at −20°C before testing. The residual inhibitory activities of samples were determined by HIV-1 NL4-3-based single-cycle infection assay, and IC_50_ values were calculated as described above.

### The *in vivo* anti-HIV-1 activity of bispecific inhibitors in rats

To evaluate the *ex vivo* antiviral activity of bispecific inhibitors, a rat model without animal infection facilities was performed as described previously (29). Briefly, bispecific inhibitors were subcutaneously administered to five male SD rats (6 weeks) at 3 mg/kg. Serum samples were collected from the orbital venous plexus at different time points: before injection (0 h) and 1 h, 4 h, 8 h, 12 h, 24 h, 2 days, 5 days, and 7 days post-injection. The inhibitory activity of the rat sera was determined by the HIV-1 NL4-3-based single-cycle infection assay described above, and 50% effective concentration was defined as the serum dilution fold that inhibited 50% virus infection.

## Data Availability

Data will be made available upon reasonable request.

## SUPPLEMENTAL MATERIAL

Supplemental figures jvi.02018-24-s0001.docx Figures S1 to S3.

ASM does not own the copyrights to Supplemental Material that may be linked to, or accessed through, an article. The authors have granted ASM a non-exclusive, world-wide license to publish the Supplemental Material files. Please contact the corresponding author directly for reuse.
