# Supplementary material for: Addition of a short HIV-1 fusion-inhibitory peptide to PRO 140 antibody dramatically increases its antiviral breadth and potency
Source: J Virol. 2025 Mar 25;99(4):e02018-24. doi: 10.1128/jvi.02018-24 (PMC11998511; doi:10.1128/jvi.02018-24)
Supplement: Supplemental figures — Figures S1 to S3. [file jvi.02018-24-s0001.docx]

**Supplementary Data**

**
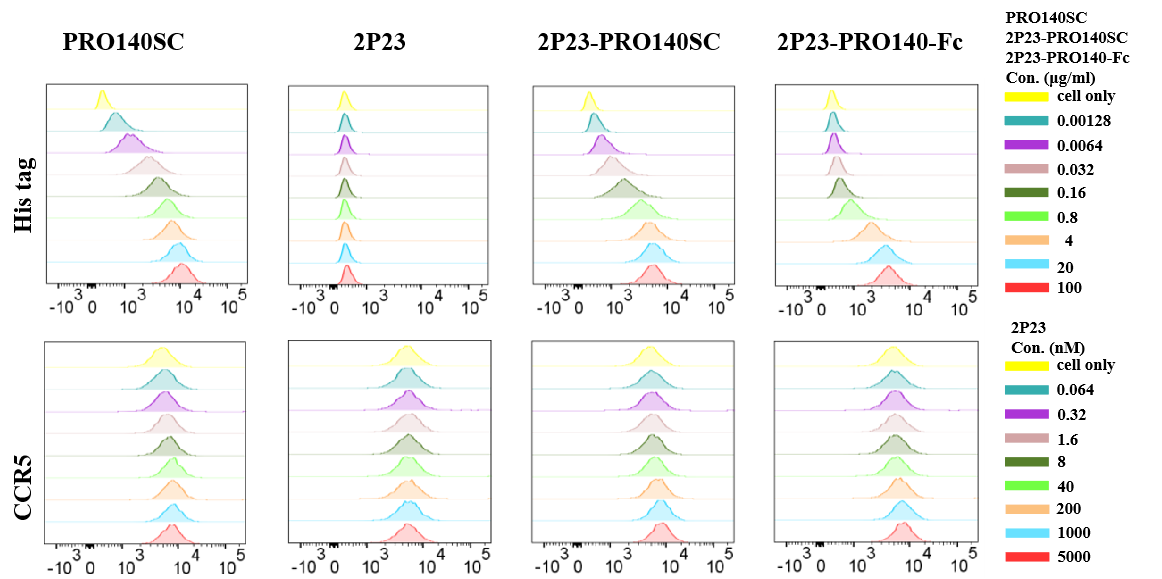
**

**Figure S1.** **Dose-dependent bindings of PRO 140-based inhibitors with TZM-bl cells. (A)** TZM-bl cells were preincubated with an inhibitor at 4℃ for 1 h, and after thorough washing, the binding ability of an inhibitor was determined by a mouse anti-His tag antibody. **(B)** The expression level of CCR5 on the cell surface was determined by amouse anti-human CD195 antibody. The fluorescence intensities of cell membrane were quantitated with a FACSCanto II instrument. Yellow, representing only TZM-bl cells without inhibitor-treated; the rest colors, representing serially diluted concentrations of inhibitor-treated cells.

**
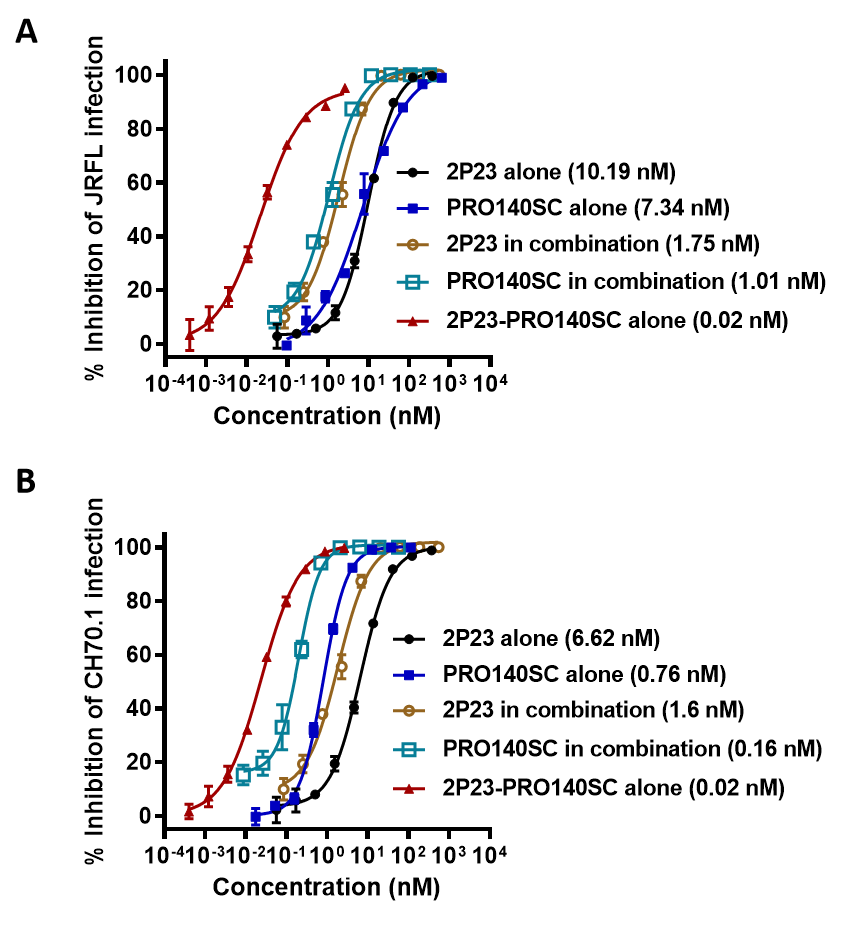
**

**Figure S2. Synergistic effects between 2P23 and PRO140SC.** The inhibitory activities of 2P23 and PRO140SC alone or in combination against HIV-1 JRFL **(A)** and CH70.1 **(B)** pseudoviruses were measured by single-cycle infection assays. The assays were performed in triplicate and repeated 3 times, and mean IC_50_ values are shown in parentheses.

**
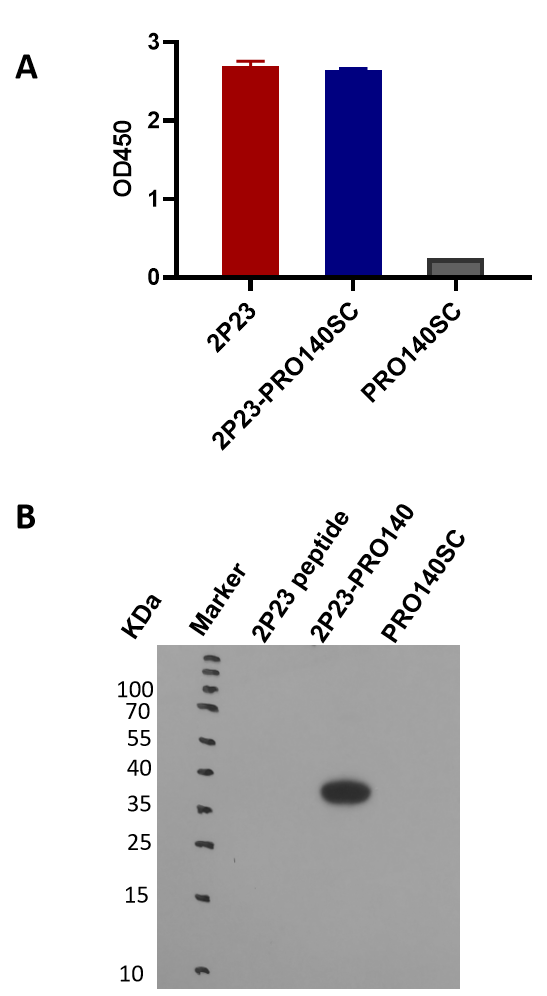
**

**Figure S3. Characterization of mouse anti-2P23 monoclonal antibody 5F7.** The reactivity of 5F7 with 2P23 peptide, 2P23-PROP140SC and PRO140SC was characterized by ELISA (A) and Western-blotting assay (B).
